# Electronic and photocatalytic properties of two-dimensional boron phosphide/SiC van der Waals heterostructure with direct type-II band alignment: a first principles study

**DOI:** 10.1039/d0ra05579d

**Published:** 2020-08-28

**Authors:** Thi-Nga Do, M. Idrees, Bin Amin, Nguyen N. Hieu, Huynh V. Phuc, Nguyen V. Hieu, Le T. Hoa, Chuong V. Nguyen

**Affiliations:** Laboratory of Magnetism and Magnetic Materials, Advanced Institute of Materials Science, Ton Duc Thang University Ho Chi Minh City VietNam dothinga@tdtu.edu.vn; Faculty of Applied Sciences, Ton Duc Thang University Ho Chi Minh City VietNam; Department of Physics, Hazara University Mansehra 21300 Pakistan; Department of Physics, Abbottabad University of Science and Technology Abbottabad 22010 Pakistan; Institute of Research and Development, Duy Tan University Da Nang 550000 Vietnam lethihoa8@duytan.edu.vn; Faculty of Natural Sciences, Duy Tan University Da Nang 550000 Vietnam; Division of Theoretical Physics, Dong Thap University Cao Lanh 870000 Vietnam; Department of Physics, The University of Da Nang, University of Science and Education Da Nang Vietnam; Department of Materials Science and Engineering, Le Quy Don Technical University Ha Noi Vietnam

## Abstract

Designing van der Waals (vdW) heterostructures of two-dimensional materials is an efficient way to realize amazing properties as well as opening opportunities for applications in solar energy conversion and nanoelectronic and optoelectronic devices. In this work, we investigate the electronic, optical, and photocatalytic properties of a boron phosphide–SiC (BP–SiC) vdW heterostructure using first-principles calculations. The relaxed configuration is obtained from the binding energies, inter-layer distance, and thermal stability. We show that the BP–SiC vdW heterostructure has a direct band gap with type-II band alignment, which separates the free electrons and holes at the interface. Furthermore, the calculated absorption spectra demonstrate that the optical properties of the BP–SiC heterostructure are enhanced compared with those of the constituent monolayers. The intensity of optical absorption can reach up to about 10^5^ cm^−1^. The band edges of the BP–SiC heterostructure are located at energetically favourable positions, indicating that the BP–SiC heterostructure is able to split water under working conditions of pH = 0–3. Our theoretical results provide not only a fascinating insight into the essential properties of the BP–SiC vdW heterostructure, but also helpful information for the experimental design of new vdW heterostructures.

## Abstract 2

We investigate the structural, electronic, optical and photocatalytic properties of boron phosphide and SiC monolayers and their corresponding van der Waals heterostructure by density functional theory.

## Graphical abstract

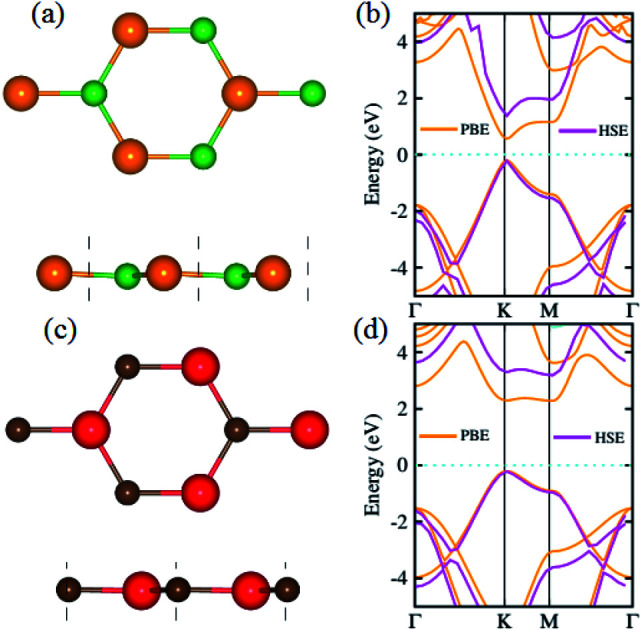

## 1 Introduction

Extremely reduced two-dimensional (2D) materials, like graphene (G), hexagonal boron nitride (BN), transition metal dichalcogenides (TMD) and Janus transition metal dichalcogenides (JTMD) are of great importance for their incredible mechanical, electronic, optical and electrochemical properties, which are appropriate for extensive applications.^1–4^ Recently, the main research interest has switched from exclusive systems to hybrid ones comprising a minimum of two chemically different 2D materials, such as graphene/BN,^5^ graphene/TMD,^6,7^ BN/TMD,^8^ graphene/silicone,^9,10^ and TMD/TMD.^11^ Such assemblies of 2D materials ultimately form 2D heterostructures, which may exhibit a wide range of attractive characteristics, such as novel electronic and optical features and enhanced effectiveness for carrier introduction by fine-tuning the barrier height.^12–16^

The honeycomb structures of BP and other group III–V binary compounds have been proposed theoretically and gained extensive attention due to their exceptional electronic properties.^17,18^ Practically, multilayer BP films have been produced from the precursor of silicon carbide under chemical vapor deposition (CVD).^19^ BP possesses a planar Young’s modulus and Poisson’s ratio of 135.6 N m^−1^ and 0.27, respectively, illustrating that its mechanical stability is similar to that of MoS_2_ and it is denser than monolayer graphene and BN. Furthermore, it exhibits a remarkable primary direct energy band gap of 0.91 eV,^17^ making it a propitious 2D material for distinctive nanoelectronics. On the other hand, the sp^2^ hybridized ZnO monolayer is prodigious in attracting research curiosity from theoretical as well as experimental approaches.^20^ The captivating physical and chemical characteristics of single-layered ZnO, including the analytic fusion and huge exciton binding energy, render this material suitable for electronic devices, photovoltaic cells, and gas sensor applications.

The creation of 2D vertical vdW heterostructures is critical for expanding the applications of condensed matter materials.^21–32^ These novel heterostructures are formed *via* vdW forces; they maintain the hallmarks of the parent components and also possess combination-induced new properties. Consequently, significant consideration has been given to this domain in order to attain modified features.^33,34^ A first principles study on the electronic characteristics of a C_2_N/MoS_2_ vertical heterostructure by Guan *et al.*^33^ showed that this material presents a staggered band position. Photogenerated holes of the C_2_N layer are easily transferred to the remnant layer of MoS_2_, leading to the separation of photogenerated charge carriers (electrons and holes). They further explored the enhanced capability of absorbing ultraviolet and visible light, which gives rise to the advanced fabrication of solar cells. Finally, the response to strain of the C_2_N/MoS_2_ heterostructure was studied, and the staggered band alignment was even conserved for certain compressive strains. Cai *et al.*^34^ conducted calculations to study the electronic properties of graphene/phosphorene (G/BP) and hexagonal boron nitride/phosphorene (hBN/BP) vdW heterostructures by density functional theory (DFT). They verified that the G/BP and BN/BP vertical heterostructures are sustained by weak bonding. Weak orbital coupling is responsible for the direct energy gap of BP when combined with G or BP, which preserves the linear dichroism, rendering it influential for adopting BN or graphene. They further observed a significant reallocation of the electrostatic potential at the junction that can remarkably renormalize the momentum of the charge carriers. Hence, electronic attributes and hole–electron dissociation can be tuned by synthesizing the vertically stacked vdW heterostructures.

Recently, careful examination of a single layer of boron phosphide confirmed its hexagonal crystal lattice, just like graphene, together with a direct energy gap, making it an extraordinary material for innovative nanoelectronic and optoelectronic device applications.^35^ Structural analysis of 2D BP and SiC showed that they both have a hexagonal lattice layout with a little lattice mismatch that ideally permits them to fabricate BP–SiC vdW heterostructures and predicts their ability for optical absorption.

In the current study, we perform computation in order to examine the distinct stacking patterns and electronic attributes of BP–SiC vdW heterostructures. We identify the most stable system through calculations of the thermodynamic stability, and also confirm the fact that the layers are held together by vdW interactions. Interestingly, BP–SiC vdW heterostructures possess direct type-II band arrangement. Furthermore, they have the capability to significantly absorb light in the visible region and can also split water at pH = 0, which makes them an important candidate for applications in the fields of photovoltaics and photocatalytic devices.

## 2 Computational details

Our calculations are based on density functional theory (DFT) with the projector augmented-wave (PAW) method^36^ as executed by the Vienna *Ab initio* Simulation Package (VASP) code.^37^ The Perdew–Burke–Ernzerhof (PBE) function^38^ and generalized gradient approximation (GGA) are manipulated to delineate the exchange and correlation of electrons.^39^ A computed cutoff energy of 500 eV is employed. The Monkhorst–Pack mesh scheme is used to determine the *k*-point estimation.^40^ Additionally, the first Brillouin zone (BZ) is illustrated by (30 × 30 × 1) for the PBE and (12 × 12 × 1) for the HSE06 calculations. The atoms are perfectly stable when the force is smaller than 0.01 eV Å^−1^. The total energy is standardized to 10^15^ eV per single atom. All the unfavorable interactions in the formation of the heterostructure are easily avoided by adjusting the vacuum level up to 25 Å along the *z*-direction. Furthermore, the Heyd–Scuseria–Ernzerhof (HSE06)^41,42^ are performed to obtain precise electronic arrangement and desirable optical attributes. Keeping the layer by layer vdW forces, Grimme’s^43^ DFT-D2 method is appropriate for all simulations, and has been verified to render a sound description for diverse vdW heterobilayer networks.^44,45^

The simulation of binding energy (*E*_b_) is given by *E*_b_ = *E*_het_ − *E*_1st_ − *E*_2nd_, where *E*_het_, *E*_1st_ and *E*_2nd_ represent the aggregate energies of the heterostructure and first and second monolayers, respectively. The redistribution of charge in the heterobilayer is easily apprehended by the plane-averaged charge density difference Δ*ρ*(*z*) along the *z*-direction perpendicular to the heterostructure Δ*ρ*(*z*) = *ρ*_H(*z*)_ − *ρ*_1stP(*z*)_ − *ρ*_2nd(*z*)_, where *ρ*_H(*z*)_ is the plane-averaged charge density of the heterostructure, and *ρ*_1stP(*z*)_ and *ρ*_2nd(*z*)_ are specified for the first and second pristine monolayers, respectively.

## 3 Results and discussion

**Fig. 1:**
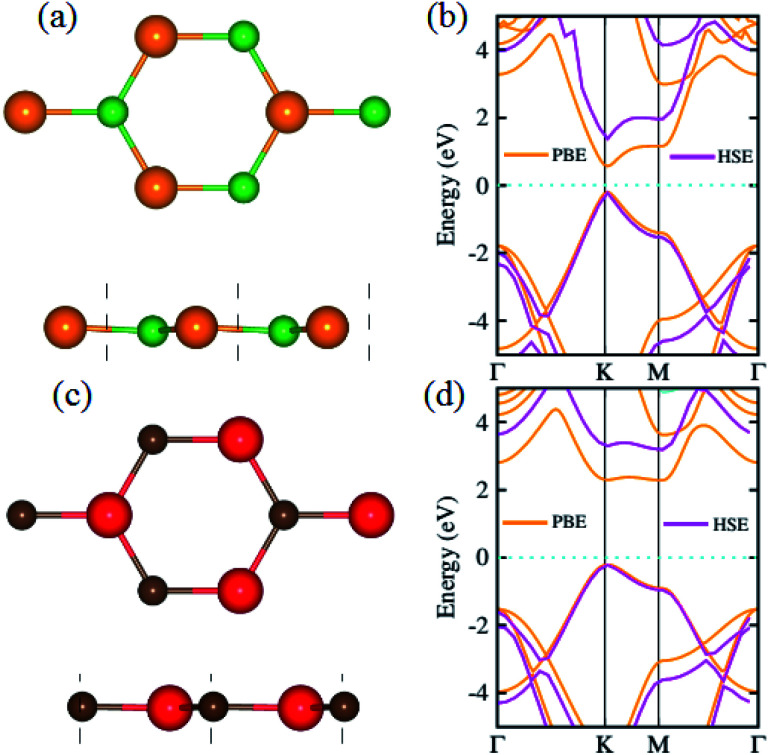
(a and c) Top, side and (b and d) band structures of BP and SiC monolayers, respectively; atoms are colored green (orange) for B (P) and red (brown) for Si (C), respectively.

We begin with the calculations of structural properties of pristine BP and SiC monolayers to confirm the precision of our computational method. Fig. 1(a–d) present the top and side views as well as the band structures of these materials. The lattice constants of pristine BP and SiC monolayers are 3.20 Å and 3.09 Å, while their band gap values are 1.63 eV (1.0 eV) and 3.40 eV (2.3 eV) using HSE (PBE), respectively. The lattice parameter of the BP–SiC heterostructure is 3.145 Å, which is the average of those for the BP and SiC monolayers. It means that the lattice parameter of the BP monolayer is compressed, but that of the SiC monolayer is stretched. Also, this demonstrates that the lattice mismatch for the BP–SiC heterostructure is small, about 1.75%, and has little influence on the properties of the BP–SiC heterostructure. The band structure of BP shows direct band nature, while that of SiC exhibits indirect band nature (see Fig. 1(b and d)). All these values are in excellent agreement with previous calculated data.^46–48^ Since the lattice constants of the BP and SiC monolayers show very small lattice mismatch, they are suitable for designing a vdW heterostructure with a precise stacking configuration. It should be noted that both boron phosphide (BP) and SiC exhibit spin-nonpolarized nonmagnetic semiconductor activity,^49^ and so does the BP–SiC heterostructure. Previously, Dong *et al.*^50^ constructed a lateral vdW heterostructure between one-dimensional (1D) zigzag boron phosphide nanoribbons (zBPNRs) and silicon carbide nanoribbons (zSiCNRs) with edges terminated by hydrogen atoms. Such edges might cause the formation of the spin-polarized magnetic states in zBPNRs, zSiCNRs and their heterostructures. This formation was also observed in zigzag graphene nanoribbons.^51^ The authors also employed a spin-unpolarized calculation to investigate the electron transport properties of the planar hybrid zSiC–BP–SiC nanoribbon devices owing to their nonmagnetic semiconductor activity. Motivated by previous studies, in this work we use a spin-unpolarized calculation to investigate the electronic and photocatalytic properties of the planar 2D vdW BP–SiC heterostructure.

We consider six possible stacking configurations of the BP–SiC vdW heterostructure: (a) B atom on top of Si atom and P atom above C atom; (b) the inverse of (a); (c) B atom on top of Si, while P and C are on the hexagonal side; (d) the inverse of (c); (e) P above C, while B and Si are on the hexagonal side; (f) the inverse of (e). We calculate the binding energies of all stackings and also their inter-layer distances to find the most stable configuration. The binding energies (inter-layer distances) of stackings (a), (b), (c), (d), (e) and (f) are −0.12 eV (3.41 Å), −0.18 eV (3.38 Å), −0.24 eV (3.19 Å), −0.20 eV (3.26 Å), −0.14 eV (3.30 Å) and −0.15 eV (3.29 Å), respectively. Our calculations show that stacking configuration (c) is the most stable, which is our focus for the further study of electronic and photocatalytic properties discussed below.

**Fig. 2:**
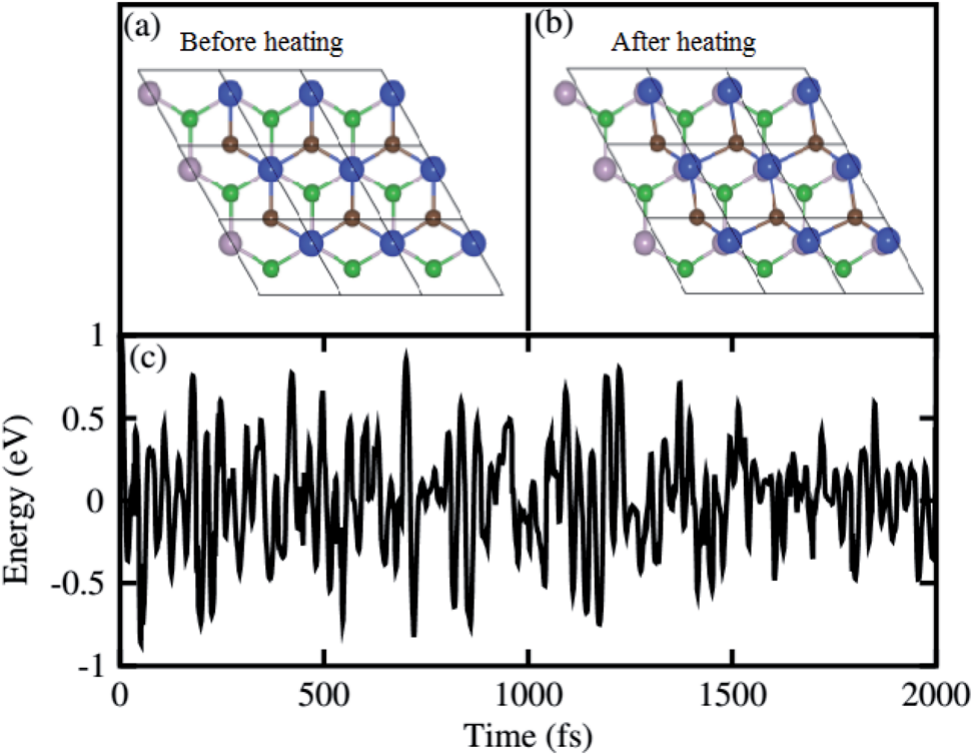
The atomic structures of BP–SiC vdW heterostructure (a) before and (b) after heating. (c) Thermal stability of BP–SiC vdW heterostructure at 300 K.

**Fig. 3:**
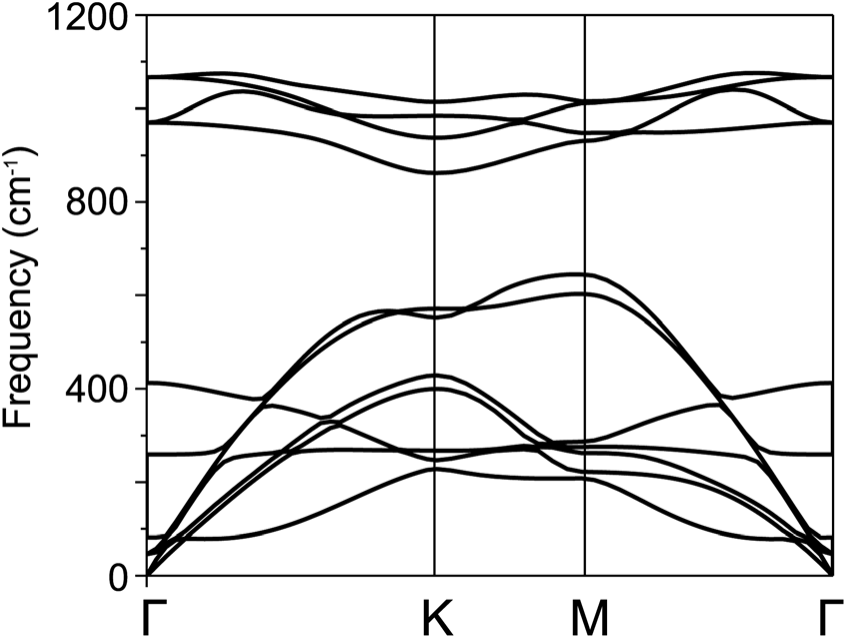
Calculated phonon dispersion curves of BP–SiC heterostructure.

For further confirmation of stability, we used *ab initio* molecular dynamics (AIMD) simulations for the BP–SiC vdW heterostructure. Fig. 2 shows that the BP–SiC vdW heterostructure retains its geometry without any structural distortion after 6 ps. Also, this system has slight variation in the total energy, implying its thermal stability at room temperature (*T* = 300 K). Furthermore, in order to verify the stability of this heterostructure at the ground state, we further calculate its phonon dispersion curves, as depicted in Fig. 3. The phonon dispersion curves show that the BP–SiC heterostructure exhibits no imaginary frequency mode, indicating its dynamic stability.

**Fig. 4:**
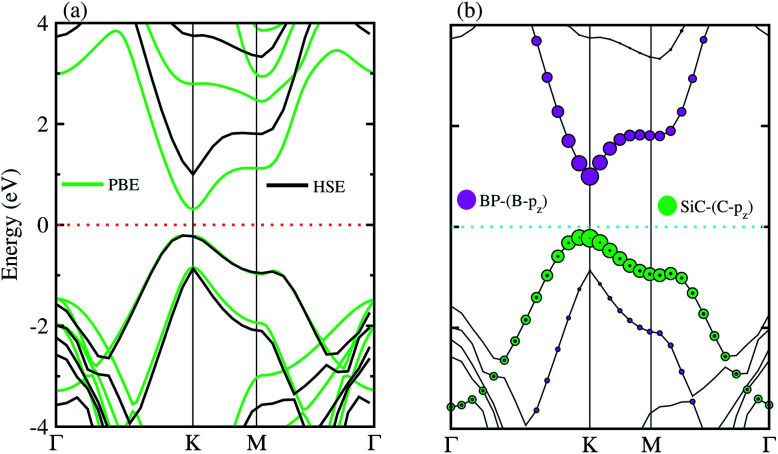
(a) Electronic and (b) weighted band structure of BP–SiC vdW heterostructure.

The electronic band structures of the BP–SiC vdW heterostructure are displayed in Fig. 4(a) using HSE and PBE calculations. Here, the green lines represent the PBE result, while the black lines show the HSE calculations. One can see that the BP–SiC vdW heterostructure shows direct band nature where the VBM and CBM are located at the K points of Brillouin zone for both PBE and HSE calculations. The direct band nature of the BP–SiC vdW heterostructure makes this material a potential candidate for the fabrication of optoelectronic devices.^52^ The band gap using PBE calculations is 0.51 eV, while it increases to 1.23 eV by using the HSE method. The band gap value of the BP–SiC vdW heterostructure is smaller than that of the pristine BP and SiC monolayers, but it is still larger than that required for photocatalysis reactions, showing the potential application of the BP–SiC vdW heterostructure as a visible light photocatalyst.^53^

**Fig. 5:**
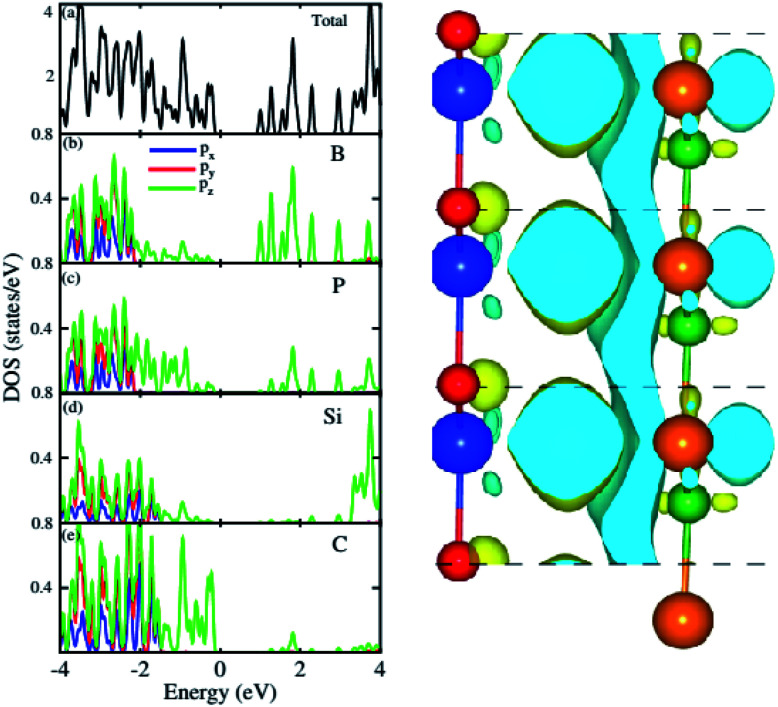
Total and partial densities of states of BP–SiC vdW heterostructure (left panel) and the charge density difference (right panel).

For further verification of type-I and type-II band alignment we have investigated the weighted band structure of the BP–SiC vdW heterostructure, as shown in Fig. 4(b). The weighted band structure is calculated by the HSE method. One can easily find that the VBM of the BP–SiC vdW heterostructure is from the C-p_*z*_ state of the SiC monolayer, while the CBM is due to the B-p_*z*_ state of the BP monolayer, hence confirming type-II band alignment. The VBM and CBM are localized from the SiC and BP monolayers, respectively, at the interface between the two layers. Such type-II band alignment spontaneously separates electrons and holes, enabling high efficiency optoelectronics and solar energy conversion.^54^ The total and partial densities of states of B, P, Si, and C atoms are plotted in Fig. 5(a–e), respectively. Our calculations show that the contribution of B and C atoms of the p_*z*_ state to the CBM and VBM is larger than that of the other states, which confirms the weighted band and type-II band alignment. Furthermore, all these orbital overlaps can modify the orbitals and enhance optical absorption.^54^ The layer localized at the CBM potentially acts as an electron donor, while the VBM acts as an electron acceptor in the corresponding heterostructures. Although an external electric field applied to homogeneous bilayers enables spatial charge separation and results in a type-II band alignment,^55^ we have avoided an electric field in the present work. The photogenerated free charge carriers are effectively separated, indicating the potential of these vdW heterostructures for prominent applications in solar energy conversion.^56,57^

**Fig. 6:**
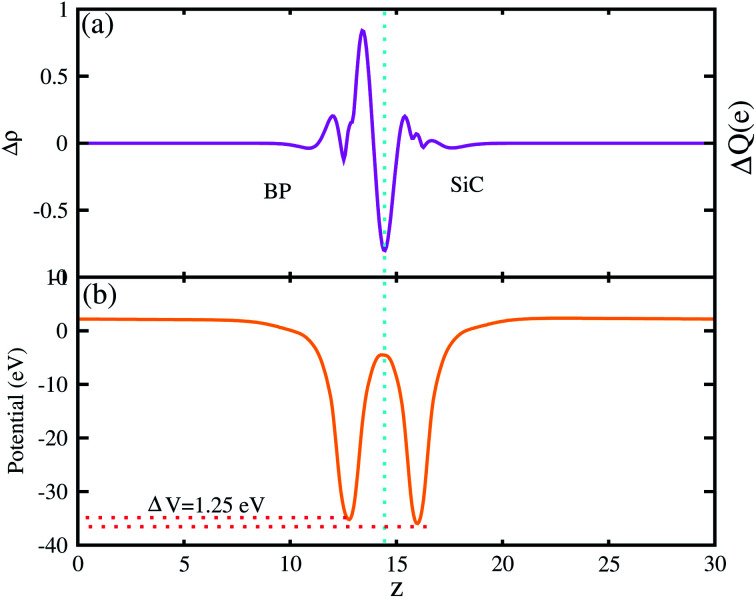
(a) Planar and (b) plane-average electrostatic potential of BP–SiC vdW heterostructure.

The 3D isosurface of the charge density difference is illustrated in Fig. 5 (right panel). The yellow and cyan areas represent electron accumulation and depletion, respectively. One can observe that charges are transferred from the BP to the SiC layer. We further investigate the planar and plane-average electrostatic potential of the BP–SiC vdW heterostructure, as presented in Fig. 6(a and b), respectively. It is clear that the BP layer donates electrons to the SiC layer, leading to p-doping in BP and n-doping in the SiC layer. The total number of electrons transferred is determined by Bader charge analysis at the BP–SiC interface. It shows that about 0.015*e* is transferred from the BP to the SiC layer.^58^Fig. 6(b) shows the plane-average electrostatic potential of the BP–SiC vdW heterostructure along the *z*-direction. It is clear that the SiC layer has deeper potential than the BP layer, driving electrons to move from the BP to the SiC layer.

In order to explore the optical properties of the BP–SiC vdW heterostructure, we calculate the imaginary part *ε*_2_(*ω*) of the dielectric function. The optical characteristics of materials can be understood through the absorption spectra. The dielectric function that estimates the response of a material to electromagnetic waves in terms of frequency, *ε*(*ω*), is expressed as *ε*(*ω*) = *ε*_1_(*ω*) + i*ε*_2_(*ω*).

**Fig. 7:**
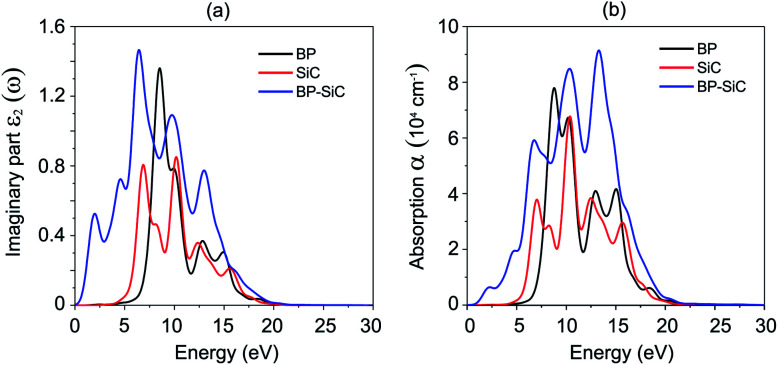
(a) Imaginary parts of dielectric functions and (b) optical absorption as a function of photon energy of BP, SiC and BP–SiC heterostructure.

The dielectric function constitutes a real part, *ε*_1_(*ω*), and an imaginary part, *ε*_2_(*ω*). Fig. 7(a) presents the imaginary parts of the dielectric functions of the heterostructure and the constituent monolayers. We find that the imaginary part of the dielectric function of the BP–SiC heterostructure is larger than that of the individual BP and SiC monolayers. There are two distinguished peaks in the ultraviolet region (above 3.11 eV) and visible light region (1.61–3.11 eV) from the imaginary part of the dielectric function, indicating enhanced optical properties of the BP–SiC heterostructure compared with the constituent monolayers. The optical absorption of BP, SiC and the BP–SiC heterostructure is depicted in Fig. 7(b). The formation of the BP–SiC heterostructure leads to an increase in the intensity of optical absorption compared to that of both the BP and SiC monolayers. The intensity of optical absorption can reach up to about 10^5^ cm^−1^. Therefore, it is obvious that the BP–SiC heterostructure acts as a high-performance absorber of sunlight, which is useful for attaining high-efficiency photocatalysts.

Current research is investigating the significance of hydrogen fuel cell technology as a pollution-free source of energy, so it is convenient to determine the capacity of materials for photocatalytic water splitting. It is perceived as a process by which water molecules decouple in a photoelectrochemical cell *via* photocatalysis. The use of water is based on its renewable and economic nature. It can be examined by valence and conduction band edge potentials (*E*_VBM_ and *E*_CBM_) that originate from Mulliken electronegativity:^59^1
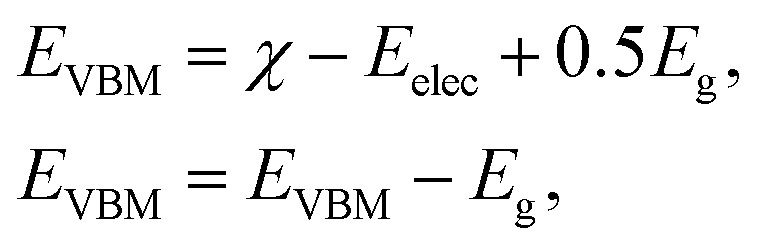
where *E*_elec_ is the standard electrode potential with a value of 4.5 V on the hydrogen scale, whereas the geometric mean of component atoms is represented by *χ*. It is 0.5 times the sum of electron affinity and first ionization energy of the individual atoms. The electron affinities of the BP and SiC monolayers and the BP–SiC heterostructure are calculated to be 1.011 eV, 1.62 eV and 1.913 eV, respectively. The calculated first ionization potentials of BP, SiC and the BP–SiC heterostructure are 9.32 eV, 9.58 eV and 9.44 eV, respectively.

**Fig. 8:**
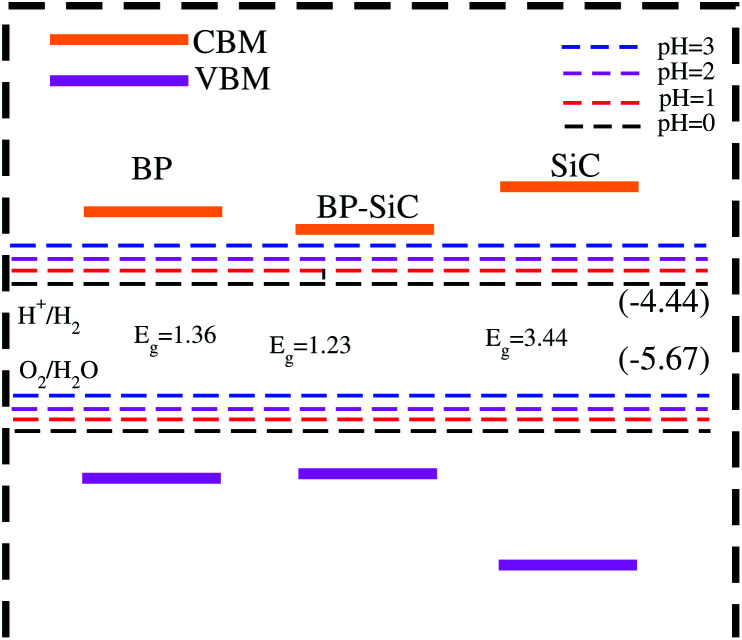
Photocatalytic response of the BP and SiC monolayers and BP–SiC vdW heterostructure.

In Fig. 8, the black lines represent the redox potentials for water splitting at pH = 0. One can observe that for both the monolayers and their corresponding heterostructure both the VB and the CB edges achieve energetically favorable positions and straddle the redox potentials and are hence suitable for water splitting at pH = 0. For the BP monolayer the CBM (VBM) values are greater than 0.65 eV (0.25 eV) from the redox potentials. Similarly, for the SiC monolayer the CBM (VBM) also gives higher values than the redox potential. In the case of the BP–SiC vdW heterostructure, these values go to the average of the corresponding monolayers and also straddle the redox potential, hence the photocatalytic properties show a good response at pH = 0. As a matter of fact, the band edges calculated using the HSE approach confirm its suitability for water splitting at pH = 0 and demonstrate that it is a highly efficient photocatalyst for the conversion of solar light into hydrogen, which is an attractive technique for applications in the production of clean and renewable energy devices.^60^ In addition, it is clear that the reduction and oxidation potentials are influenced by the pH level *via E*^red^ = −4.44 + pH × 0.059 eV and *E*^oxd^ = −5.67 eV + pH × 0.059 eV, respectively. Thus, the reduction and oxidation potentials at pH = 1, 2, 3 are also calculated for water splitting, as depicted in Fig. 8. One can find that the band edges of the BP–SiC heterostructure are located at energetically favourable positions, representing the ability of the BP–SiC heterostructure to split water under working conditions of pH = 0–3.

Finally, it should be noted that there are several common methods for the synthesis of 2D materials and their heterostructures, including mechanical or liquid-phase exfoliation and chemical vapor deposition (CVD) or epitaxial growth. For example, a graphene/MoS_2_ vdW heterostructure has been synthesized by a transfer procedure.^61^ Theoretically, Shu *et al.* predicted that a liquid-transfer technique can be used to fabricate a vdW heterostructure based on GaN and BP materials.^62^ Thus, the BP–SiC heterostructure can also be synthesized in experiments by a transfer method and molecular beam epitaxy.

## 4 Conclusion

In summary, we have investigated the structural, electronic, optical and photocatalytic properties of BP and SiC monolayers and their corresponding heterostructure by using density functional theory. The BP–SiC vdW heterostructure shows a direct type-II band alignment, while the BP (SiC) monolayers have direct (indirect) band nature. In the BP–SiC vdW heterostructure the VBM and CBM are from different monolayers, hence potentially separate the free electrons and holes. Bader charge analysis shows that the BP layer donates electrons to the SiC layer, making BP p-doping while SiC is n-doping. The imaginary part of the dielectric function and optical absorption of the BP–SiC heterostructure are enhanced compared with those of the constituent monolayers. The intensity of optical absorption can reach up to about 10^5^ cm^−1^. Therefore, it is obvious that the BP–SiC heterostructure acts as a high-performance absorber of sunlight, which is useful for attaining high-efficiency photocatalysts. The band edges of the BP–SiC heterostructure are located at energetically favourable positions, indicating that the BP–SiC heterostructure is able to split water under working conditions of pH = 0–3. Therefore, we conclude that the BP–SiC vdW heterostructure is a promising material for optical, photovoltaic and photocatalytic devices.

## Conflicts of interest

The authors declare that there are no conflicts of interest regarding the publication of this paper.

## Supplementary Material
